# Does International Travel Frequency Affect COVID-19 Biosecurity Behavior in the United States?

**DOI:** 10.3390/ijerph18084111

**Published:** 2021-04-13

**Authors:** Myung Ja Kim, C. Michael Hall, Mark Bonn

**Affiliations:** 1 The College of Hotel & Tourism Management, Kyung Hee University, Seoul 02447, Korea; 2 Department of Management, Marketing, and Entrepreneurship, University of Canterbury, Christchurch 8140, New Zealand; michael.hall@canterbury.ac.nz; 3 Geography Research Unit, University of Oulu, 90014 Oulu, Finland; 4 Ekonomihögskolan, Linnéuniversitet, Universitetskajen, Landgången 6, 39182 Kalmar, Sweden; 5 Department of Service Management and Service Studies, Lund University, Campus Helsingborg, 25108 Helsingborg, Sweden; 6 Dedman School of Hospitality & Tourism Management, Florida State University, Tallahassee, FL 32306-2541, USA; mbonn@dedman.fsu.edu

**Keywords:** COVID-19, biosecurity, international travel frequency, market segmentation, Value–Attitude–Behavior theory, the United States

## Abstract

High-quality biosecurity practices are critical to restarting international tourism. Effective market segmentation improves the communication and efficacy of health advice. Travel frequency is an important basis for health-related consumer segmentation, as it is closely related to risk of greater exposure to infectious diseases. Theoretically grounded studies of tourist biosecurity behavior and travel frequency have largely been neglected, although insights into practices and attitudes are especially relevant for coronavirus disease of 2019 (COVID-19 (coronavirus disease of 2019) health responses. Therefore, this research constructed and tested a conceptual model applying Value–Attitude–Behavior theory to US travelers to see whether the frequency of international travel affected tourist COVID-19 related biosecurity behavior. US respondents were drawn from a panel using a quota sampling technique according to the age and gender of American outbound tourists. An online survey was administered in September 2020. The responses (n = 395) of those who traveled internationally within five years were analyzed utilizing partial least squares-structural equation modeling (PLS-SEM) with multi-group analysis. Travel frequency significantly affects biosecurity behavior. High travel frequency (≥8 trips) has the strongest effect of value on biosecurity attitudes, personal norms, social norms, and biosecurity social norms, leading to biosecurity behaviors. Biosecurity behaviors pertaining to medium travel frequency (4–7 trips) are significantly influenced by personal norms. At low travel frequency (1–3 trips) levels, biosecurity behaviors are stimulated by biosecurity attitudes and social norms, showing the highest predictive power among the three groups. This work provides insights into international travel consumer biosecurity practices and behavior. From a market segmentation perspective, the levels of international travel frequency have various influences on biosecurity values, attitudes, personal norms, social norms, and behaviors. The biosecurity behaviors of low-frequency travelers are found to be the most significant of the three groups, suggesting that individuals who travel less frequently are more likely to practice responsible COVID-19 biosecurity behavior.

## 1. Introduction

Greater human mobility, driven by growth in air travel, is a leading factor in the increased reach of infectious diseases (e.g., COVID-19 (coronavirus disease of 2019), MERS-CoV (Middle East respiratory syndrome), Zika virus) [1,2,3]. Biosecurity can be defined as a range of specific intervention measures that have been put in place by national and regional governments along with pre-existing border biosecurity requirements for tourism and trade to restrict the spread of infectious diseases [1]. Reducing biosecurity risks is a significant issue for tourism given its role as a vector in biological invasion and transfer, suggesting appropriate travel guidelines [4,5,6]. Therefore, understanding what influences international tourist’s biosecurity behavior is valuable and timely, particularly in relation to the frequency of international travel by individuals, which may increase the risk of acquiring and transmitting infectious disease during outbreaks [4,5,6,7].

The travel and tourism sector has been dramatically impacted by COVID-19 [8]. Since reducing travel mobility and congregation (for events, meetings, and hospitality) are standard non-pharmaceutical interventions to restrict the spread of transmissible disease, COVID-19 has disproportionately and deeply affected the tourism sector [9,10]. Nevertheless, high-quality biosecurity practices are critical to restarting international tourism both for reducing the potential for contagion and to improve consumer confidence in traveling to destinations during and/or in the post COVID-19 pandemic [11]. Furthermore, the recovery of the tourism, hospitality, and visitor economy sectors is greatly affected by how tourists’ modify their biosecurity behaviors to meet governmental and destination requirement. Therefore, an improved understanding of tourist biosecurity behavior in relation to COVID-19 would seem fundamental [1,12,13].

Effective market segmentation improves the communication and efficacy of health advice [14,15], particularly in terms of COVID-19 biosecurity behavior [1]. Travel frequency is an important basis for health-related consumer segmentation, as it is closely related to the risk of greater exposure to infectious diseases, along with levels of perception of risk by travelers [4,5,6,7,16,17,18], suggesting the need for a better understanding tourists’ biosecurity practices [13].

Value–Attitude–Behavior (VAB) theory is a well-established explanatory framework used in health marketing based on a systematic review and meta-analysis [19]. In a tourism context, research on tourists’ values has shown how these influence attitudes, including personal norms and social norms, which in turn lead to travel consumers’ behaviors [20]. VAB theory indicates that peoples’ values with respect to, for example, environmentally friendly consumption has an impact on their attitude, personal norm, and social norm relevant to their behavior in relation to waste reduction in tourism-related contexts [21]. Individuals’ values and attitudes influence their behavioral response to COVID-19 public health measures, such as mask wearing and compliance with rules [22]. However, theoretically grounded studies have largely been ignored in relation to tourist biosecurity behavior, travel frequency, and VAB theory, suggesting insights into tourist biosecurity practices and attitudes that are especially relevant for COVID-19 health responses. In order to fill this gap, the purpose of this study was to construct and test a conceptually integrated model with respect to tourist COVID-19 biosecurity behavior applying the VAB model and three frequency groups of overseas travel (1–3, 4–7, ≥8 international trips) using the analytics of partial least squares-structural equation modeling (PLS-SEM) [23].

The structure of this paper is as follows. Section 2 describes the theoretical background and hypotheses development as well as includes the literature review. Section 3 discusses materials and methods, and Section 4 analyzes the results. Finally, Section 5 summarizes the discussion with the final section providing the conclusions and limitations of this work.

## 2. Literature Review

### 2.1. Theoretical Background

#### 2.1.1. Biosecurity and Tourism

Biosecurity refers to “the protection of a country or region, or a location’s or firm’s economic, environmental, and/or human health from harmful organisms” ([24], p. 121). From a tourism perspective, biosecurity strategies can be applied at different stages of the trip cycle: decision-making and anticipation, travel to a tourism destination or attraction, the on-site experience, return travel, and recollection of the experience [3,25]. From a medical tourism perspective, biosecurity is a real concern in terms of disease transmission, health care access, and health system readiness [26,27,28]. Tourists and tourism infrastructure can act as a vector for the introduction of invasive alien species (IAS) and disease, representing substantial biosecurity risk for tourism destinations worldwide [29,30]. Air travel can rapidly connect any two points on the planet, and this has the potential to cause swift and broad dissemination of emerging and reemerging infectious diseases that may pose a threat to global health security [2]. In particular, tourism-related biosecurity behavior is essential during a pandemic [1]. Accordingly, this study considers biosecurity behavior as a key factor among international travel consumers.

#### 2.1.2. Market Segmentation by Travel Frequency

Researchers have been interested in tourism market segmentation from a variety of perspectives [1,14,15,31,32,33,34]. In terms of destination image, four market segments identified as cultural explorer, specialty enthusiast, natural seeker, and family devotee show a significant difference in frequencies of travel and the average expenditure on accommodation per night [32]. Four segments of festival attendees identified as locals, highly involved enthusiasts, first timers/nonloyals, and fringe attendees reveal significant differences in terms of number of times attended, distance from the event, length of trip, likelihood to return, expenditures per person, average age, and income [33]. Effective market segmentation for travel frequency improves the communication and efficacy of health advice, particularly during disease outbreaks [1,2,6,7,14,15,34]. Despite its potential importance, little research has conducted on market segmentation with respect to the frequency of international travel; therefore, this study attempts to examine market segments on travel frequency of overseas tourists as high, medium, and low groups in the context of COVID-19.

#### 2.1.3. Value–Attitude–Behavior

VAB theory has been applied to explain the relationships among individuals’ health value, attitude, and/or behavior, including in relation to health information technologies, gender differences, and healthy food choices [19,22,35]. Studies have highly predicted consumer behavior utilizing the VAB theory in the context of sustainable tourist practices, showing that values have impacts on attitudes, personal norms, and social norms that influence behaviors [20,21,36,37,38]. Tourism researchers have widely utilized the VAB model to better understand the relationships between tourists’ values, attitudes, and behaviors, showing that values influence attitudes, which in turn lead to behaviors [39,40,41]. Even though the VAB theory is significant in health and tourism research, further opportunities exist to better understand the role of the VAB theory in biosecurity and health-related tourisms, including in the context of the COVID-19 pandemic.

### 2.2. Hypotheses Development

A value can be defined as an enduring belief that a specific mode of conduct or end-state is personally preferable to its opposite [42]. Values have been shown to influence attitudes relevant to behaviors in sustainability contexts [43]. Medical tourists’ attitudes derived from values can be defined as a predictor of behaviors that constitute the final phase in the VAB hierarchy [43,44]. In the health tourism setting, consumer value is the key element that inspires their attitude toward healthy practices [35]. Drawing upon the literature review above, the hypothesis for three frequency groups is suggested as follows:

**Hypothesis** **1** **(H1).** *Biosecurity values have a positive effect on biosecurity attitudes for travel during the COVID-19 pandemic in high, medium, and low-level groups of travel frequency.*

Personal norm refers to an individual’s sense of moral obligation to conduct a particular action; thus, the behavioral relevance of a personal norm is limited to actions containing a moral dimension [20,21,45]. Values on sustainable consumerism have highly significant influence on personal norms on sustainability crowdfunding [45]. Values on environmentally friendly consumerism positively influence healthy eating for the planet [21]. Values on eco-tourism lead to personal norms among cruises [20]. Based on the literature review above, the hypothesis for three frequency groups are suggested as follows:

**Hypothesis** **2** **(H2).** *Biosecurity values have a positive effect on biosecurity personal norms for travel during the COVID-19 pandemic in high-, medium-, and low-level groups of travel frequency.*

Social norm, which is interchangeably utilized with the term subjective norm in the extant literature, indicates an individual’s perceived level of the social pressure to conduct or not to conduct a particular action in a specific situation [20,21,45]. From the perspectives of sustainability and tourism, values are a key antecedent of social norms [20,21,45]. According to the literature, the hypothesis for three frequency groups are suggested as follows:

**Hypothesis** **3** **(H3).** *Biosecurity values have a positive effect on biosecurity social norms for travel during the COVID-19 pandemic in high-, medium-, and low-level groups of travel frequency.*

Tourist biosecurity behavior can be defined as practices to prevent the transfer of infectious diseases, such as COVID-19, or exotic flora and fauna between locations during travel [1]. Risk attitude toward COVID-19 has a negative effect on travel intention [46]. Individual attitudes toward COVID-19 restriction measures lead to behaviors such as wearing face masks [22]. During the COVID-19 pandemic, attitude towards international travel has a significant effect on short- and long-term avoidance behavior [16]. In line with the literature review above, we suggest the following hypothesis:

**Hypothesis** **4** **(H4).** *Biosecurity attitudes have a positive effect on tourist biosecurity behavior during the COVID-19 pandemic in high-, medium-, and low-level groups of travel frequency.*

From an eco-friendly tourism perspective, potential tourists’ personal norm has been shown to have a highly positive impact on their behavioral intention, such as word-of-mouth intention, buying intention, and intention to sacrifice [20]. In sustainable consumerism, personal norms are a key antecedent of behaviors for environmentally friendly consumptions [21]. Furthermore, sustainable crowdfunders’ personal norm leads to their participation in sustainability consumerism practices [45]. In association with the literature, the authors anticipate the following hypothesis:

**Hypothesis** **5** **(H5).** *Biosecurity personal norms have a positive effect on tourist biosecurity behavior during the COVID-19 pandemic in high-, medium-, and low-level groups of travel frequency.*

Regarding user acceptance of consumer-oriented health information technologies, users’ social norms (e.g., subjective norms) have positive influences on their behavioral intention to use information technologies [19]. Social interactions on walking and cycling are strongly associated with a higher use of active transport [41]. Consumers’ social norms on sustainability significantly lead to their behaviors of sustainable practices [20,21,45]. In compliance with the literature, this research posits the following hypothesis:

**Hypothesis** **6** **(H6).** *Biosecurity social norms have a positive effect on tourist biosecurity behavior during the COVID-19 pandemic in high-, medium-, and low-level groups of travel frequency.*

## 3. Materials and Methods

This study applied prior validated multi-measurement questions which were reworded to fit the study context [47]. Data were collected via an online survey consisting of 25 items in order to measure five constructs, including biosecurity values, biosecurity attitudes, biosecurity personal norms, biosecurity social norms, and tourist biosecurity behavior. Items relevant to biosecurity values (six questions), biosecurity attitudes (three questions), biosecurity personal norms (three questions), and biosecurity social norms (three questions) were based on the existing literature [20,21,22,45]. Each representative statement of values, attitudes, personal norms, and social norms read as follows: “Supporting plant biosecurity is a virtuous behavior when traveling,” “Participating in travel-related biosecurity is a positive behavior,” “I feel an obligation to participate in travel-related biosecurity,” and “Most people who are important to me think I should participate in travel-related biosecurity at any time.” Tourist biosecurity behavior was assessed using 10 questions formed from previous studies [1,3,5,9], with an example statement being: “When I travel, I always make sure that my shoes are clean and have no dirt on the soles.”

Three university professors who are experts in biosecurity and/or tourism conducted an evaluation of content validity. After this step, four questions related to tourist biosecurity behavior when traveling were added to better capture the concept (i.e., “When traveling, I keep away from people with a cough or runny nose,” “I usually wear a face mask when traveling in planes or public transport,” “I frequently wash my hands when I travel,” and “When I travel, I always cover my mouth and nose with a tissue when I sneeze”). These questions were also developed in light of advice gained from the application of non-pharmaceutical interventions during pandemics [48]. In addition, three online survey professionals assessed if the survey could suitably evaluate international travel behavior. Instructions, general questions, and socio-demographic variables were also revised to fit the online survey system based on the professions’ comments. Moreover, the polit test was conducted on three Ph.D. students. According to the results of the polit test, the question items on the five constructs are improved to better communicate with respondents. A pre-test was subsequently administrated to 40 U.S. residents who had previously traveled overseas during the prior five years period. Based upon the pre-test, two questions about guaranteeing the quality of survey data and time spent for answering all items were added. At this stage, minor changes were also made to the tourist biosecurity behavior questions (see Appendix A).

As a result of the ability to obtain responses cost-effectively and rapidly, especially when employing a large panel, online surveys have been frequently applied for research [49]. Given the contingencies of the COVID-19 pandemic, an online survey was also regarded as being appropriate for health and safety purposes. This study utilized the online survey firm Qualtrics, who possesses one of the world’s largest panels as well as following and adhering to rigorous procedures for collecting valid data [50]. American respondents were drawn from a Qualtrics panel based on a quota sampling technique according to the age (18 and over) and gender of outbound tourists based on data from the US National Travel and Tourism Office [51]. All respondents were asked two screening questions with regard to commitment to providing thoughtful and honest answers and overseas trip experience. Scaled questions were rotated to help avoid response bias so that every respondent received different orders of items. The online survey was administrated on 1–5 September 2020. From 411 respondents, seven respondents who finished the questionnaire in less than four minutes and nine respondents who did not undertake overseas travel in the past five years were eliminated. In addition, outliers and inappropriate responses were excluded from the dataset by analyzing normal distributions and exploring data based on frequencies, descriptives, p-p plots, and correlations. Thus, a total of 395 responses were analyzed utilizing PLS-SEM with multi-group analysis [52], indicating that they had previously traveled internationally within the five years and wanted to continue traveling internationally when COVID-19 is over.

PLS-SEM was employed to estimate the current research framework. PLS-SEM is useful in estimating first-order constructs concurrently with formative second-order constructs [23]. Additionally, PLS-SEM is better than typical SEM (e.g., covariance based) for non-normal data, small samples, and/or for complicated models with multi-group analysis (MGA) [53]. For these reasons, this study utilized SmartPLS 3.2.3 to validate the measurement and structural models [52]. To verify the moderating effect of low and high Big Five personality groups, the researchers also used MGA according to PLS-SEM algorithms [54].

## 4. Results

Growth in the frequency of overseas travel, including air tourism, has contributed to the spread of infectious diseases [4,6,7]. However, travel consumers’ behaviors are different depending on their levels of travel frequencies [4,6,7,33,34]. Moreover, travel frequency explains a variety of consumer travel behaviors [34]. Accordingly, based upon international travel frequencies of United States residents over the most recent 5-year period, three travel segments were created and named: the high (eight or more trips; 126 cases; mean = 22.92), medium (four to seven trips; 115 cases; mean = 4.98), and low (one to three times; 154 cases; 2.14) travel groups (Table 1). Regarding demographics and general questions, sample profiles of the three frequency groups are provided in detail (Table 2). Thus, comparing three groups are statically appropriate in terms of mean differences, characteristics, and sample sizes of three groups.

With the PLS approach, a minimum sample size of 100 with six hypotheses appears best to balance the trade-offs for detection and accurate estimate, which strives for the reliability possible in the measures [54]. In the PLS-SEM, larger sample sizes (>100 cases) are generally preferable, although smaller sample size (<100) are acceptable depending on the context of the research [23]. Moreover, the sample size in PLS can be greater than 10 times the maximum numbers of inner or outer model links pointing at any latent variable [53]. Accordingly, the sample sizes of high, medium, and low-frequency groups in this study are statistically acceptable for the proposed research model with utilizing PLS-SEM.

According to confirmatory factor analysis (CFA), 22 items had factor loadings greater than 0.7, and three items with factor loadings below 0.7 were removed (see Table 3). The composite reliability, Cronbach’s α, and Rho_A (reliability coefficient) of constructs were above 0.7, approving the internal consistency validity [53]. The average variance extracted (AVE) of variables was above 0.5, and the factor loadings of items were above 0.7, approving the convergent validity (Table 4). All the corrections in the five constructs were statistically significant, all AVEs were greater than 0.5, and the square root of AVEs was greater than each correlation coefficient, thus supporting discriminant validity [52]. Moreover, Q^2^ values above zero were found for all endogenous constructs, suggesting acceptable levels of predictive relevance. Finally, the standardized root mean residual (SRMR) of model fit is 0.086, which is lower than the cutoff of 0.9.

**Figure 1:**
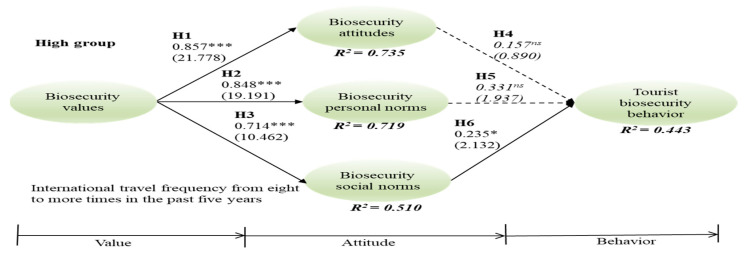
High group of international travel frequency. *** *p* < 0.001; * *p* < 0.5; n s = non-significant.

**Figure 2:**
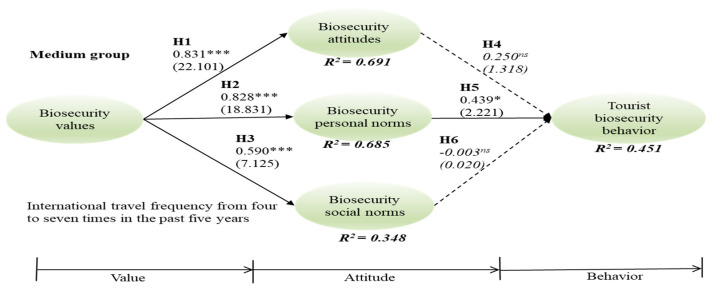
Medium group of international travel frequency. *** *p* < 0.001; * *p* < 0.5; ns = non-significant.

**Figure 3:**
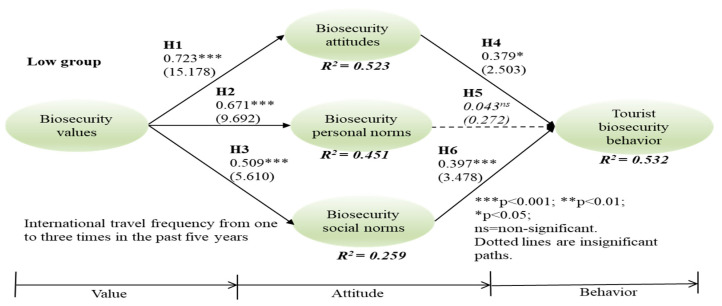
Low group of international travel frequency. *** *p* < 0.001; * *p* < 0.5; ns = non-significant.

Since the data had non-normal distributions by both skewness and kurtosis (see Table 3), this study utilized PLS-SEM to assess the six hypotheses for three groups, applying bootstraps of 5000 re-sampling techniques. In the high-frequency group, relationships between biosecurity value and attitude (γ = 0.857, t = 21.778, *p* < 0.001), value and personal norms (γ = 0.848, t = 19.191, *p* < 0.001), value and social norms (γ = 0.714, t = 10.462, *p* < 0.001), and social norms and behavior (β = 0.235, t = 2.132, *p* < 0.05) were significant; thus, H1, H2, H3, and H6 were supported. In the medium group, relationships between biosecurity value and attitude (γ = 0.831, t = 21.101, *p* < 0.001), value and personal norms (γ = 0.828, t = 18.831, *p* < 0.001), value and social norms (γ = 0.590, t = 7.125, *p* < 0.001), and personal norms and behavior (β = 0.439, t = 2.221, *p* < 0.05) are significant, supporting H1, H2, H3, and H5. In the low group, relationships between biosecurity value and attitude (γ = 0.723, t = 15.178, *p* < 0.001), value and personal norms (γ = 0.671, t = 9.692, *p* < 0.001), value and social norms (γ = 0.509, t = 5.610, *p* < 0.001), attitude and behavior (β = 0.379, t = 2.503, *p* < 0.05), and social norms and behavior (β = 0.397, t = 3.478, *p* < 0.001) are significant, supporting H1, H2, H3, H5, and H6 (Figure 1, Figure 2 and Figure 3).

## 5. Discussion

Results reveal that biosecurity values have significant effects on biosecurity attitudes, personal norms, and social norms, which influence tourist biosecurity behavior in all three groups of international travelers from America, therefore supporting the relevance of VAB theory in describing US international tourist biosecurity behaviors. The results are consistent with the previous findings on the VAB model in the context of tourism and sustainability [20,21,45]. The high frequency of the international travel group has the strongest influence of biosecurity values on the VAB model, followed by the medium and low groups, inferring that using levels of international travel frequency is significant in predicting likely biosecurity attitudes, personal norms, social norms, and tourist biosecurity behavior. Given the important role of international travel in the spread of infectious diseases, including in the context of COVID-19, this research provides further insights into international tourism management practices [46,55] and improvements in biosafety and biosecurity in responding to contagious diseases [56].

From a market segmentation perspective based on travel frequency, the levels of international travel frequency have various influences on biosecurity values, attitudes, personal norms, social norms, and behaviors in the USA. The findings are similar to the prior research on differences depending on levels of travel frequencies [4,6,7,33,34]. The biosecurity behaviors of high-frequency tourists are the least significant (R^2^ = 0.443), and the biosecurity behaviors of low-frequency travelers are the most significant among the three groups (R^2^ = 0.532), suggesting that individuals who travel less frequently are more likely to better practice COVID-19 biosecurity behaviors. These results may also potentially reflect the perceived familiarity of frequent fliers with biosecurity measures, which may contribute to a false sense of security and level of biosecurity knowledge when traveling internationally.

## 6. Conclusions

The results of this work suggest several contributions to better understanding tourism-related biosecurity behavior, especially in the post-pandemic travel environment. First, in applying the VAB theory, this research sheds light on biosecurity behavior when traveling during the COVID-19 pandemic, extending prior studies on responsible tourism behaviors and sustainability consumerism [20,21,45]. Second, based on the market segment of travel frequency, the three groups of high, medium, and low show substantial differences with respect to biosecurity behavior practices, significantly expanding past literature on the differences of travel frequencies and diseases spreading during outbreaks [1,2,6,7,14,15,34]. Third, biosecurity behavior practices are also significantly affected by attitude, followed by personal norm and social norm, expanding the literature between attitude toward international travel and behavior [16], between social norm and behavior for sustainability consumerism [45], and social norms and sustainable behavior [20,21].

This study has practical implications for public policy makers for the development of more effective marketing communication strategies to international tourists. In order to encourage overseas travelers to practice appropriate biosecurity behaviors, airlines as well as health and border agencies should focus on enhancing positive attitudes toward biosecurity, which is the strong predictor in the model. In addition, international travel frequency is a useful segmentation tool to improve the targeting of travel health messages and reducing undesirable behavior [57]. Thus, it is suggested that policy makers could promote their messages on tourist biosecurity practices through a range of different social media and online or mobile communication channels, suggesting that participation in travel-related biosecurity is constructive, beneficial, and essential, since current tourists massively use the internet and social networks [58]. If international and national health organizations want to target the biosecurity practices of high-frequency travelers, for example via frequent flier programs, they should concentrate on the social norms (e.g., subjective norms) of that group. In contrast, personal norm messaging appears more suitable for influencing medium-frequency international traveler behavior relevant to tourist biosecurity behavior. When low-frequency travelers are targeted, the focus should be on biosecurity attitudes and social norms in order to increase their compliance with biosecurity requirements associated with tourist biosecurity practices.

## 7. Limitations and Future Research Directions

Even though this study has provided insights in terms of tourist biosecurity behaviors during outbreaks, several limits are identified, which can be opportunities of future research. This survey was conducted in the US during a highly politicized period of the COVID-19 pandemic, so caution needs to be applied in generalizing the findings to other countries, cultures, and contexts. Since this study has focused on tourist biosecurity practices during the COVID-19 pandemic, future research may be conducted when the impacts of the pandemic on consumer behavior for biosecurity have abated. Since the online surveys in this study were analyzed by traditional statistical approaches, further study would be interesting when crawling data from social media and applying big data analytics and artificial intelligence analysis. Future segmentation research on differences in traveler characteristics and the implications that they have for biosecurity practices would be valuable for the development of appropriate social and health marketing communications to reduce biosecurity risks.

## Figures and Tables

**Table 1:** Grouping three groups of international travel.

Group	Frequency Range	Sample Size	Mean
High	8 and more times	126	22.92
Medium	4–7 times	115	4.98
Low	1–3 times	154	2.14

**Table 2:** Demographic characteristic of the high, medium, and low-frequency groups of international travel.

Characteristics	High (%)	Medium (%)	Low (%)	Characteristics	High (%)	Medium (%)	Low (%)
**Gender**				**Monthly household income**			
Male	69.0	46.1	34.4	Less than US$2000–39,999	19.1	28.7	42.9
Female	31.0	53.9	64.3	From US$4000 to 7999	27.8	38.3	36.4
Other	0.0	0.0	1.3	US$8000 or more	53.1	33.0	20.8
**Age**				**Overseas travel intent if COVID-19 ends**			
Between 18 and 29 years old	19.0	31.4	37.1	Yes	99.2	94.8	91.6
Between 30 and 39 years old	31.8	19.1	16.2	No	0.8	5.2	8.4
Between 40 and 49 years old	32.6	13.9	9.7	**Overseas travel frequency in the past 5 yeas**			
Between 50 and 59 years old	9.5	13.0	18.8	8 times and over (high group: 126 cases)	100	0.0	0.0
60 years old and over	7.1	22.6	18.2	4–7 times (medium group: 115 cases)	0.0	100	0.0
**Educational level**				1–3 times (low group: 154 cases)	0.0	0.0	100
Less than or high school diploma	7.1	8.7	15.6	**Had COVID-19**			
2-year college	8.7	20.9	26.6	Yes	12.7	7.0	9.7
University	29.4	32.2	39.0	No	87.3	93.0	90.3
Graduate school or higher	54.8	38.3	18.8	**Know someone who had COVID-19**			
**Marital status**				Yes	54.0	58.3	52.6
Single	19.8	33.0	44.2	No	46.0	41.7	47.4
Married	79.4	64.4	47.4	**Cancel a trip than wear masks**			
Divorce, widow/er, living together	0.8	2.6	8.4	Yes	32.5	38.3	37.0
**Occupation**				No	67.5	61.7	63.0
Professional (e.g., attorney, engineer)	36.5	33.0	23.5	**Cancel a trip than enter quarantine**			
Business owner/self-employed	11.1	13.0	11.7	Yes	58.7	60.0	66.2
Service worker	13.5	7.0	12.3	No	41.3	40.0	33.8
Office/administrative/clerical worker	11.9	8.7	14.3	**Residential area**			
Civil servant (government)	0.8	5.2	1.9	Northeast	46.0	33.8	26.0
Home maker	2.4	3.5	1.9	South	27.8	34.8	38.9
Student	5.6	4.3	9.1	Midwest	10.4	15.8	18.9
Retiree	5.6	14.8	15.6	West	15.0	15.6	15.0
Unemployed	2.4	5.2	3.2	Alaska	0.8	0.0	0.6
Other (e.g., flight attendant, chief executive officer)	10.3	5.2	6.5	Hawaii	0.0	0.0	0.6

**Table 3:** Confirmatory factor analysis (CFA) and descriptive statistics.

Constructs	Factor Loading	Mean	VIF **	Kurtosis	Skewness
**Biosecurity values**					
1. Supporting plant biosecurity is a virtuous behavior when traveling.	0.738	5.458	2.050	0.626	*−1.029*
2. Practicing animal biosecurity is a moral duty when traveling.	0.772	5.430	2.130	0.996	*−1.164*
3. Participating in human biosecurity is an ethically right action when traveling.	0.795	5.532	2.289	0.786	*−1.130*
4. Wearing a mask helps biosecurity when traveling.	0.844	5.592	3.055	*1.202*	*−1.329*
5. Social or physical distancing contributes to biosecurity when traveling.	0.857	5.618	3.018	*1.310*	*−1.328*
6. Quarantine assists biosecurity when traveling.	0.813	5.484	2.694	0.959	*−1.169*
**Biosecurity attitudes**					
1. Participating in travel-related biosecurity is a positive behavior.	0.921	5.691	3.125	*1.825*	*−1.391*
2. Participating in travel-related biosecurity is a beneficial behavior.	0.930	5.651	3.403	*1.186*	*−1.225*
3. Participating in travel-related biosecurity is an essential behavior.	0.927	5.676	3.244	*1.282*	*−1.241*
**Biosecurity personal norms**					
1. I feel an obligation to participate in travel-related biosecurity.	0.908	5.628	2.722	*1.236*	*−1.284*
2. Regardless of what other people do, because of my own values/principles, I feel that I should participate in travel-related biosecurity.	0.921	5.635	3.040	*1.337*	*−1.296*
3. I feel that it is important to participate in travel-related biosecurity for reasons of sustainability.	0.919	5.610	2.955	*1.548*	*−1.315*
**Biosecurity social norms**					
1. Most people who are important to me think I should participate in travel-related biosecurity at any time.	0.903	5.481	2.526	0.646	*−1.012*
2. Most people who are important to me would want me to participate in travel-related biosecurity at any time.	0.902	5.473	2.450	0.938	*−1.143*
3. Most people who are important to me support my participation in travel-related biosecurity at any time.	0.888	5.608	2.332	0.694	*−1.025*
**Tourist biosecurity behavior**					
1. When I travel, I always make sure that my shoes are clean and have no dirt on the soles. *	-	-	-	-	-
2. When I travel, I always make sure that my clothes are clean.	0.791	5.790	1.997	*1.336*	*−1.327*
3. When I travel, I always make sure that my bags are clean and have no dirt or seeds on them.	0.666	5.580	1.497	0.901	*−1.123*
4. When I travel, I never carry food to another country. *	-	-	-	-	-
5. When I travel, I always make sure I fill in any customs or agricultural declaration form correctly.	0.795	5.997	2.022	*3.102*	*−1.695*
6. When I travel, I always find out what I can or cannot take into another country before I get there.	0.772	6.048	1.858	*2.925*	*−1.728*
7. When traveling, I keep away from people with a cough or runny nose.	0.775	5.734	1.938	*1.626*	*−1.373*
8. I usually wear a face mask when traveling in planes or public transport. *	-	-	-	-	-
9. I frequently wash my hands when I travel.	0.810	6.086	2.225	*2.904*	*−1.738*
10. When I travel, I always cover my mouth and nose with a tissue when I sneeze.	0.805	5.818	2.096	*1.612*	*−1.362*

Note: * Items are deleted after CFA. The items in italics have non-normal distribution. ** Variance inflation factor of multicollinearity.

**Table 4:** Reliability and discriminant validity.

Construct	Correlation of the Constructs				
	1	2	3	4	5
1. Biosecurity values	**0.804**				
2. Biosecurity attitudes	0.793 **	**0.926**			
3. Biosecurity personal norms	0.772 **	0.847 **	**0.916**		
4. Biosecurity social norms	0.600 **	0.684 **	0.701 **	**0.898**	
5. Tourist biosecurity behavior	0.578 **	0.628 **	0.628 **	0.585 **	**0.775**
Cronbach’s alpha ≥ 0.7	0.890	0.917	0.904	0.880	0.888
Rho_A (reliability coefficient) ≥ 0.7	0.895	0.917	0.904	0.881	0.892
Composite reliability ≥ 0.7	0.916	0.947	0.940	0.926	0.913
AVE ≥ 0.5	0.647	0.857	0.839	0.806	0.600
Effect size (Q^2^) > 0		0.534	0.495	0.286	0.269
SRMR of model fit: 0.086 < 0.09					

Note: All boldfaced diagonal elements appearing in the correlation of constructs matrix indicate the square roots of AVEs. ** Correlation is significant at the 0.01 level (2-tailed).

## Data Availability

The data presented in this study are available on request from the corresponding author. The data are not publicly available due to ethical reasons.

## Appendix A

Questionnaire. A survey on biosecurity and tourism

**We care about the quality of our survey data and hope to receive the most accurate measures of your opinions, so it is important to us that you thoughtfully provide your best answer to each question in the survey.**

**Do you commit to providing your thoughtful and honest answers to the questions in this survey?**

1. I will provide my best answers: Go to the next question.

2. I will not provide my best answers: End the survey.

3. I can’t promise either way: End the survey.

**Screen question (SQ)**

SQ1. Have you ever traveled internationally?

① Yes ☞ If you checked “yes,” please answer the following GQ1 question.

② No: Close the survey (We thank you for your time spent taking this survey.

Your response has been recorded.).

**General question (GQ)**

GQ1. Do you plan to travel internationally if the pandemic ends?

① Yes ② No

GQ2. How many times have you traveled internationally in the past 5 years?

__________________times

GQ3. Did/do you have COVID-19?

① Yes ② No

GQ4. Do you know someone who have/had COVID-19?

① Yes ② No

GQ5. Would you rather cancel a trip than wear masks?

① Yes ② No

GQ6. Would you rather cancel a trip than enter quarantine?

① Yes ② No

**Note 1: Biosecurity** is the protection of the economic, environmental, and/or human health in a country, region, or location from the introduction, emergence, establishment, and spread of harmful organisms (pests and diseases). In this study, **biosecurity** refers to measures that are taken to stop the spread or introduction of organisms potentially harmful to human, animal, and plant life. The main aim of biosecurity is to protect human health, agriculture, forestry, fishing, and the environment through the prevention, control, and management of biological risk factors, such as the introduction of plant or animal pests, or a disease (e.g., COVID-19).

**Note 2:** In this study, **travel, traveling, tourism, and tourists** mean ***international*** travel, traveling, tourism, and tourists.

**Construct question (CQ)**

CQ1. Please carefully read each item and check the score that you think best fits [Select one for each] (1: strongly disagree; 2: disagree; 3: somewhat disagree; 4: neither agree nor disagree; 5: somewhat agree; 6: agree; 7: strongly agree).

**CQ1. Biosecurity values**	Strongly disagree	Dis- agree	Somewhat disagree	Neither agree nor disagree	Somewhat agree	Agree	Strongly agree
1. Supporting plant biosecurity is a virtuous behavior when traveling.	1	2	3	4	5	6	7
2. Practicing animal biosecurity is a moral duty when traveling.	1	2	3	4	5	6	7
3. Participating in human biosecurity is an ethically right action when traveling.	1	2	3	4	5	6	7
5. Wearing a mask helps biosecurity when traveling.	1	2	3	4	5	6	7
6. Social or physical distancing contributes to biosecurity when traveling.	1	2	3	4	5	6	7
7. Quarantine assists biosecurity when traveling.	1	2	3	4	5	6	7

**CQ2. Biosecurity attitudes**	Strongly disagree	Dis- agree	Somewhat disagree	Neither agree nor disagree	Somewhat agree	Agree	Strongly agree
1. Participating in travel-related biosecurity is a positive behavior.	1	2	3	4	5	6	7
2. Participating in travel-related biosecurity is a beneficial behavior.	1	2	3	4	5	6	7
3. Participating in travel-related biosecurity is an essential behavior.	1	2	3	4	5	6	7

**CQ3. Biosecurity personal norms**	Strongly disagree	Dis- agree	Somewhat disagree	Neither agree nor disagree	Somewhat agree	Agree	Strongly agree
1. I feel an obligation to participate in travel-related biosecurity.	1	2	3	4	5	6	7
2. Regardless of what other people do, because of my own values/principles, I feel that I should participate in travel-related biosecurity.	1	2	3	4	5	6	7
3. I feel that it is important to participate in travel-related biosecurity for reasons of sustainability.	1	2	3	4	5	6	7

**CQ4. Biosecurity social norms**	Strongly disagree	Dis- agree	Somewhat disagree	Neither agree nor disagree	Somewhat agree	Agree	Strongly agree
1. Most people who are important to me think I should participate in travel-related biosecurity at any time.	1	2	3	4	5	6	7
2. Most people who are important to me would want me to participate in travel-related biosecurity at any time.	1	2	3	4	5	6	7
3. Most people who are important to me support my participation in travel-related biosecurity at any time.	1	2	3	4	5	6	7

**CQ5. Tourist biosecurity behavior**	Strongly disagree	Dis- agree	Somewhat disagree	Neither agree nor disagree	Somewhat agree	Agree	Strongly agree
1. When I travel, I always make sure that my shoes are clean and have no dirt on the soles.	1	2	3	4	5	6	7
2. When I travel, I always make sure that my clothes are clean.	1	2	3	4	5	6	7
3. When I travel, I always make sure that my bags are clean and have no dirt or seeds on them.	1	2	3	4	5	6	7
4. When I travel I never carry food to another country	1	2	3	4	5	6	7
5. When I travel, I always make sure I fill in any customs or agricultural declaration form correctly.	1	2	3	4	5	6	7
6. When I travel, I always find out what I can or cannot take into another country before I get there.	1	2	3	4	5	6	7
7. When traveling, I keep away from people with a cough or runny nose.	1	2	3	4	5	6	7
8. I usually wear a face mask when traveling in planes or public transport.	1	2	3	4	5	6	7
9. I frequently wash my hands when I travel.	1	2	3	4	5	6	7
10. When I travel, I always cover my mouth and nose with a tissue when I sneeze.	1	2	3	4	5	6	7

**Demographic characteristics (DQ)**

DQ1. What is your gender?

① Male ② Female ③ Other

DQ2. What is your age?

① Under 20 years old

② Between 20 and 29 years old

③ Between 30 and 39 years old

④ Between 40 and 49 years old

⑤ Between 50 and 59 years old

⑥ 60 years old and over

DQ3. What is the highest level of education you have completed?

① High school diploma or lower ② 2-year college attending or degree

③ 4-year university attending or degree ④ Graduate school attending or degree

DQ4. What is your marital status?

① Single ② Married ③ Other (specify) _____

DQ5. What is your monthly household income?

① Less than US$2000 ② US$2000–3999 ③ US$4000–5999 ④ US$6000–7999 ⑤ US$ 8000 or more

DQ6. What is your occupation?

① Professional (e.g., attorney, engineer, architect) ②Entrepreneur/Self-employed ③ Service employee ④ Office/Administrative/Clerical ⑤ Civil Servant (Government) ⑥ Home maker ⑦ Student ⑧ Retiree ⑨ Unemployment ⑩ Other (specify)_______

DQ7. In what state do you normally reside?

__________________

We thank you for your time spent taking this survey.

Your response time has been recorded!
